# Development of cancer support services for patients and their close ones from the Cancer Society of Finland’s perspective

**DOI:** 10.1080/17482631.2021.1915737

**Published:** 2021-04-21

**Authors:** Heli Tiirola, Veli-Matti Poutanen, Riitta Vornanen, Liisa Pylkkänen

**Affiliations:** a Department of Social Sciences, Faculty of Social Sciences and Business Studies, University of Eastern Finland, Kuopio, Finland; b Clinical Oncology, Department of Oncology, University of Turku, Turku, Finland

**Keywords:** Cancer, care pathway, support, counselling, Cancer Society of Finland

## Abstract

**Purpose**: This study examined what support cancer patients and their close ones need and how this support should be organized when developing cancer care pathways. The study focused on the opinions of professionals of the Cancer Society of Finland (CSF), who play a central role in presenting the third sector’s perspective on care pathways.

**Method**: Six semi-structured group interviews were carried out with counselling nurses (n = 12) and managers (n = 9) of the CSF during summer 2017. The results were analysed using content analysis.

**Results**: Both patients and their close ones need more information, psychosocial support and financial counselling after diagnosis, during rehabilitation and follow-up, at relapse and during the palliative care phase; additionally, close ones require support after the patient’s death. Participants emphasized close collaboration between public healthcare and the CSF to meet the needs of patients and their close ones.

**Conclusion**: Psychosocial support can—and should—be provided as part of the care pathway. This support can be provided by organizations in the third sector, such as the CSF, which have resources in this area.

## Introduction

About 35,000 people are diagnosed with cancer every year in Finland, and the Finnish Cancer Registry estimates that there will be 43,000 new cancer diagnoses in the country by 2030 (https://www.cancerregistry.fi/). At the same time, the challenges that municipalities face in covering healthcare costs are increasing—a situation that will not improve due to Finland’s ageing population and rapidly falling birth rate.

While the quality of medical treatment is high in Finland, psychosocial care for cancer patients and their close ones remains underdeveloped. Thus, there is a need to develop care pathways and support services for both groups. As a patient’s close ones and caregivers often act as the patient’s most important emotional supporters, development of support for the patient’s close ones is also important. Developing patient care pathways and strengthening the roles of third-sector organizations in patient care are among the key goals of the renewal of Finland’s social and healthcare system (Brax, 2018). This includes streamlining patient care pathways in order to increase out-patient care and reduce hospital stays. This foreseeable change increases the need for information and support for both patients and their close ones, and here the third sector could participate.

Treatment of cancer patients in Finland is guided by national and international treatment guidelines. Regional care pathways for cancer patients have been developed for different hospitals and districts, and they define the responsibilities of different actors. However, the care pathways and rehabilitation of cancer patients are still not optimal. This has been stated in other countries as well, and according to an international policy report (Wait et al., 2017), inefficiencies can exist at the system, institution and individual levels and support for caregivers is poor.

Integrated, patient-centred care should be coordinated across professionals and support systems, and it should be based on the patients’ needs and preferences. At present, there is a need to improve integrated care by focusing on care transitions, out-patient care and palliative care (Evans et al., 2015). There is also a need to identify gaps in patient care and a need for innovation and a systematic set of priorities (Aapro et al., 2017). When developing care pathways, it is also important to understand the views of different target groups (Wait et al., 2017). Further, the international cancer community emphasizes the value of patient engagement in the development of cancer care programmes and policy efforts. In this context, the role of third-sector organizations is significant in encouraging patients and laypeople to improve care systems and pathways and, thus, conditions for patients (Schear et al., 2015).

### Previous studies

Previous studies involving cancer patients have shown that timely information (i.e., information about the disease, treatment and financial assistance and various forms of support are important at different stages of the cancer trajectory (Hartzler et al., 2016; Newby et al., 2015; Zebrack et al., 2007). Close ones and caregivers, who often act as a patient’s most important emotional supporters, may experience stress (Gustavsson-Lilius, 2010; Teixeira & Pereira, 2013), emphasizing the importance of recognizing the link between anxiety and depression among those who are close to the patient (Jacobs et al., 2017). There are also studies showing that close ones need more psychosocial support and information about the disease than the patients themselves (Gustavsson-Lilius, 2010; Niemelä et al., 2010).

In Finland, relatively little research has investigated the experiences and support needs of cancer patients’ close ones. Studies have focused on the experiences of mental health professionals who use structured family-centred interventions to support the children of cancer patients (Niemelä et al., 2010), quality of life of patients with breast cancer (Salonen, 2011), perceptions of electronic social support (Yli-Uotila, 2017) and marital relations and health-related quality of life questions in patients with prostate cancer and their spouses (Harju et al., 2018). Lehto-Järnstedt et al. (2004), Lehto et al. (2015, 2018) have performed research, for example, on cancer patients’ social support. Research in Finland from the perspective of cancer organizations remains limited.

International research on the experiences and support needs of close ones has increased, focusing, for example, on caregivers (Teixeira & Pereira, 2013), peer support groups (Landry-Dattée et al., 2016), spouses (Pauwels et al., 2012), the support needs of children (Bultmann et al., 2014; Wong et al., 2010), patients with hereditary cancer (Rudkjøbing et al., 2015; Vodermaier & Stanton, 2012) and palliative care patients (Brazil et al., 2014; Lundberg et al., 2013). However, there are many people who have no family or whose close ones do not provide support.

Previous studies have structured care pathways during the stages of screening, diagnosis, specialized care, follow-up and (if needed) palliative care (Wait et al., 2017). According to Khan et al. (2017), there is increasing emphasis on the use of care plans to guide the organization and delivery of care for cancer patients. However, researchers are still in the early stages of identifying components and key facilitators of integrated care plan uptake. We were unable to identify a single integrated care plan spanning all stages of the care pathway from diagnosis to survivorship or palliative care. Basically, there is information about the kinds of support patients and their close ones would need, but in practice, the resources either focus on clinical elements of care (Bao et al., 2016) or are targeted at individual stages of the cancer trajectory (Mayer et al., 2015). Information about activites and roles of different organisations appear to be in general fragmented. However, the use of care plans in general, across various diseases and settings, has helped to reduce in-hospital complications (Rotter et al., 2010), enhanced communication between providers and improved the quality and efficiency of care (Vanhaecht et al., 2010).

### Aim of the study

Even though many support services are well established in several sectors, the roles of third-sector actors should be clarified, particularly in the development of patient-centric healthcare—that is, in amplifying the patient’s voice. The focus of this research was on finding new insights to develop cancer support services and to strengthen and streamline the role of the third sector, including cancer societies.

Cancer Society of Finland (CSF) is an umbrella organization, which covers the whole country with its 12 regional and 6 patient organizations, with about 200 professionals and 3,500 volunteers. CSF is a non-profit organization, which funding is based on donations and national competitive funding sources for different projects. It is a strong actor in the cancer support sector. Organizations under the CSF umbrella operate alongside and in collaboration with the public sector and provide information and support for cancer patients as well as their close ones. They provide rehabilitation and psychosocial support, crisis therapy, peer support and support-person services. They also provide support during palliative and end-of-life care and for close ones after the patient’s death. In addition, they offer art therapy, physical exercise, nature experiences and themed events. Services are provided according to needs, either as face-to-face meetings or via phone, email or chat though different portals.

Our goal was not to develop formal care pathways but to explore how the CSF could support public healthcare according to the needs of cancer patients and their close ones. Professionals in the third sector, such as the CSF, have deep insight into the needs of patients and their close ones and problems within the public care system. However, the perceptions of CSF professionals have not been studied in detail in Finland before.

The aim of this study was to investigate what kind of support and information cancer patients and their close ones need and how cancer care pathways should be developed from the perspective of the Cancer Society of Finland (CSF). Additional aim was to investigate how collaboration between the CSF and public healthcare organizations could be developed.

The research questions were as follows:

1. What kind of support do cancer patients and their close ones need during the different phases of the cancer trajectory from the perspective of professionals at the CSF?
2. How should cancer care pathways for patients and support services for close ones be developed to better integrate the CSF’s services into those of public healthcare?

## Materials and methods

### Participants

The aim was to recruit a representative sample of experts working at cancer societies in Finland under the CSF umbrella. To select participants, two key selection criteria were applied: the principle of maximum variability and the principle of homogeneity (Patton, 1990, p. 171, 182–183). This was done by inviting key experts from various societies of the CSF for interviews.

Assisting in the selection of key personnel was a supervisor who, through her recommendations, was able to ensure the representation of a variety of general managers of cancer societies. Individuals were emailed with an invitation to participate in the study, and all but one participated. Counselling nurses from three different cancer societies across Finland were also asked to participate in interviews. Their supervisors promised to arrange the interviews. All counselling nurses agreed.

### Data collection

Finally, 12 counselling nurses from three different organizations (3 interviews) and nine general managers (3 interviews) of different organizations were interviewed once. For professional details, see Participant characteristics in result section.

The main researcher served as moderator (Julius & Waterfield, 2019). The interviews were conducted in Finnish as semi-structured group interviews and included four main themes: (1) care pathways, (2) specialist care and counselling, (3) economic troubles and (4) co-operation between public and non-public organizations. For selection of questions we utilized previous studies on the different stages of the illness and forms of support received and needed (Girgis et al., 2011; Teixeira & Pereira, 2013; Turner et al., 2013). All interviews were performed in a consistent manner. To ensure that everyone was familiar with the interview environment, the counselling nurses’ interviews were conducted at their workplaces and interviews with general managers were conducted at the head office of the CSF. The interviewer tried to make the interview situation relaxed, which was very successful, because the discussions were open and lively.

The duration of each interview was 60–90 minutes. Transcription of the interviews was done by a professional editor. The transcript consisted of a total of 112 pages: 58.5 pages of counselling nurse interviews and 53.5 pages of manager interviews.

### Data analysis

The interviews were analysed by means of theory-based content analysis. The data analysis commenced with a data-based approach, but towards the end of the analysis, it proceeded in a theoretical way, combining the research results with earlier theory (Elo & Kyngäs, 2008). Heli Tiirola conducted the analysis and Veli-Matti Poutanen and Liisa Pylkkänen commented on it. Initially, the transcribed interviews were systematically read several times, as it was important to get a good overall picture of the collected material. Next, words, phrases and repeated themes were underlined with different coloured pencils while taking note of the material’s themes. This was done to all of the material.

The analysis proceeded on a data-driven basis with the aim of creating an abstract description of the empirical data. During the analyses the answers were read, reduced and then merged into categories of different levels of abstraction. The analysis was then returned to the research questions and topics corresponding to these questions were selected. (Elo & Kyngäs, 2008)

Subsequently, the material was transformed into subcategories (the lowest level category) relating to upper level categories, which were merged into the main categories (the highest level category). The following main categories were identified: the support needed by those diagnosed with cancer and their close ones; counselling concerning referral to special professionals (such as psychologists, social workers, physiotherapists etc.); support needs of patients and close ones at different stages of the care pathway; and development of co-operation between cancer societies and the public sector.

Interpretation of the categorized data is the final step of the content analysis. We present a summary of the research results and suggest a new path of care on the basis of the interviewed professionals views.

### Ethical issues

This study adopted good scientific practices, including care and honesty, from the research design to data preservation. The study followed the principles of the Finnish National Ethical Board (Finnish National Board on Research Integrity TENK, 2019), which indicated that since the study would not directly target patients or close ones, it did not require the opinion of the Research Ethics Committee. Issues addressed in the study, such as ideas for better forms of support, are not sensitive topics and do not interfere with human integrity. The organizations in which the participants worked had approved their participation in study and the participants were informed that they were allowed to participate in interviews. Non-participation did not affect the treatment of employees (Fouka & Mantzorou, 2011). The study protocol included obtaining informed consent. Participation in the study was voluntary and participants had the right to terminate their participation at any time. The participants received in-depth information about the implementation of the study as well as the opportunity to discuss with researchers any matter related to the study.

Analysis of the data and compilation of the results were carried out according to strict principles of research ethics including obscuring any identifiable data. All information collected in the study is confidential and handled in accordance with the Personal Data Act (523/1999, current Data protection Act 1050/2018). There were no physical, mental, chemical or other risks involved in participating in the study. The participants were also informed of the possibility of discussing participation after the study, which no one requested.

## Results

### Participant characteristics

The participating healthcare professionals were counselling nurses and were all female and aged 27–61 years at the time of the interviews. They were nurses from their background education and most had acquired further education, such as specialist nurse or occupational nurse. Some of them also had a special competence in cancer nursing. The counselling nurses had been working at cancer organizations, on average, for 10 years, ranging from less than a year to 22 years. All of them had worked previously in public healthcare and the majority for several years in cancer care. They worked at three different organizations with different catchment areas.

The general managers of regional and disease-specific cancer societies were selected from different parts of Finland to obtain a representative cross section. Six of the interviewed managers were females and three were males ranging from 39–58 years of age. They all, except one, had a university degree; many had healthcare education, but some also had economics or varied educational backgrounds. The managers had been working in their current positions, on average, for four years, ranging from a few months to eight years; a proportion of them had worked in different positions in cancer organizations prior to their current position.

Overall, both healthcare professionals and managers were experienced and had been working with cancer patients approximately 16 years and within cancer organizations 9 years.

### Professional views on the support needs of cancer patients and their close ones

According to counselling nurses and managers, people diagnosed with cancer and their close ones are generally satisfied with the medical treatment they receive, although patients and close ones do not receive enough psychosocial support, and there are insufficient opportunities for discussion with healthcare professionals in hospitals. According to participants, patients and their close ones would need clear information on cancer and its treatments as well as information concerning psychosocial reactions and support needs throughout the cancer trajectory, and they should also be encouraged to share their thoughts and feelings about cancer.

Participants also indicated that financial challenges due to cancer have increased. Patients often do not know what kinds of financial benefits they are entitled to. Information about these benefits often comes too late, such as during rehabilitation courses, which occur late in the cancer trajectory and in which only a minority of patients participate. Managers spoke about the importance of healthcare professionals’ ability to identify persons in challenging economic situations and the importance of referring them to social workers or to Finland’s social insurance institution, Kela, to discuss potential financial support and benefits. The CSF and some organizations under its umbrella also provide limited financial support for cancer patients in the form of a non-recurring financial contribution. This kind of support is currently needed more often than in the past. However, managers emphasize that providing financial support is the government’s responsibility, as the CSF has very limited financial resources.

Professional and peer support and advice on other services, including appointments with a psychologist or a social worker, were considered important, along with information on rehabilitation services. Those diagnosed with cancer and their close ones often feel the need to meet special professionals, such as social workers and occupational therapists. It can be of the utmost importance to assess the individual needs of each patient and include the patient’s close ones in this process. Many people do not seek expert help because they are not aware that it is available. Therefore, participants said that expert services should be better and more clearly integrated into care pathways.

Manager: “… the cycle of care should include the consultation and conversation services of psychologists, psychiatrists, occupational therapists, as part of which the mental wellbeing of the individual and their relatives is assessed.”

Counselling nurses considered it important to take into account the individual needs of cancer patients, which is not always done. In addition to the cancer diagnosis, other crises may arise and the person may also need to discuss these. It is fundamentally important to identify the individual needs of each person and provide timely support. To do so, mapping of needs and coordination of support are required.

According to participants, the support of close ones is very important for cancer patients. Further, close ones may be more concerned and need more support than the cancer patients themselves. These kinds of situations can place an extra burden on both patients and their close ones. According to our study, cancer patients can perceive the diagnosis and its consequences differently from their close ones and have different coping strategies. The timing of support needs may also be different for patients and their close ones. For example, the patient may not want to speak about cancer, whereas close ones would like to share their thoughts with others, such as friends.

Manager: “Situations like these, as a relative, when you somehow end up there, when these are developing, the needs of the relative are probably very close to the patient’s needs, and these kinds of reactions—very often the relative is panicking more than the patient.”

### Better marketing of services and collaboration with public healthcare

Many patients and their close ones would have needed CSF services much earlier, according to participants of this study. Some cancer societies of the CSF do have the resources to serve more clients, but the key problem is that those diagnosed with cancer, their close ones and professionals within public healthcare do not know about the services that the CSF can offer, or they have limited or incorrect ideas about the quality of services or qualifications of the service providers at the CSF. It is necessary to note that many people need different forms of support, and public healthcare does not have the resources to provide them in a timely manner. Moreover, in most cases, public healthcare cannot serve close ones, only patients.

Although participants advertise CSF services, more accurate marketing should be directed both at patients and their close ones, and to public healthcare. To bolster this effort, new marketing channels should be developed. One essential and anticipated change is that professionals within public healthcare would refer patients and their close ones to the CSF more often. However, some counselling nurses said that public healthcare professionals can even consider the competencies of the CSF a threat.

Counselling nurses emphasized that actors in the public sector should provide information about these services both in written and verbal forms, and not only after the diagnosis but also throughout the cancer care pathway. By doing so, people would be able to decide for themselves whether and when they need these services.

Manager: “And the information must be given at the right time, and in fact it can be said that it has to be repeated, it’s not enough that you give one leaflet at the stage when they learn about having cancer; that’s the diagnosis phase. The point is to continue that along the path. And then, some take advantage of this; some need those services, others don’t need them.”

The role of managers of cancer organizations is of vital importance in developing new processes and fostering collaboration between the public sector and the CSF. Regarding collaboration between the public sector and the CSF, themes of clarification of roles and improvement of information sharing surfaced in the interviews. Regular meetings and common campaigns were proposed as concrete steps towards these goals. In addition, strengthened collaboration between the CSF and healthcare and social education organizations was proposed to increase awareness of the CSF services during education. Information technology could also be utilized more in service coordination.

Participants indicated that collaboration between the CSF and public healthcare has improved and become closer in recent years, despite the geographical differences that would threaten the quality and quantity of this collaboration. Participants strongly expressed their hope that collaboration with public healthcare and the role of the CSF would be further strengthened and clarified throughout the cancer trajectory. Only in this way could the CSF’s resources be best directed to those in need of services. They also proposed that CSF managers, in collaboration with public healthcare professionals, should define the roles and positions of CSF professionals throughout the cancer trajectory. Thus, a clear process should be developed that leads to the advent of policy-level strategy and steering mechanisms which integrate the third sector into the new structures of social and healthcare.

### Support needs for patients and their close ones throughout the cancer trajectory

Participants identified several phases of the cancer care pathway during which support is very much needed. These different phases are discussed separately.

### Support during the diagnosis phase

Any suspicion of cancer is a great shock for most people. During this phase, in which diagnostic procedures can take several weeks, support from public healthcare is limited or even non-existent. The CSF professionals can provide information, advise on and discuss support during that phase.

Counselling nurse: “And then when you think about the phase when you start the tests and they suspect that you might have cancer, you don’t have the kind of support network at this point; you’re at the testing phase. So, we could give support at that point because waiting is hard.”

The CSF can provide psychosocial support and information for both patients and close ones even as early as before diagnosis, during the period when cancer is only suspected. Some people may need only peer-group support, which cancer professionals recommend from the diagnosis phase. In this study, the treatment phase did not receive any kind of prominence; rather, the focus was on targeting CSF services to patients and their close ones at the time of diagnosis.

### Support during follow-up and rehabilitation

At the end of cancer treatment, when patients are transitioning to the follow-up and rehabilitation phase, they and their close ones generally expect a return to normal life including the resumption of their other normal roles. Psychological reactions during this phase can be surprisingly strong and both counselling nurses and managers spoke a lot about support services during this phase. If the CSF could support public healthcare particularly at this time, this could also produce financial savings.

According to participants, a portion of patients feel abandoned without any services. Treatment visits are usually dramatically reduced to infrequent control visits. At the same time, the psychological reactions of these patients can be strong, and they may have a strong need to discuss their feelings. Some patients and their close ones can be surprised and embarrassed by their psychological reactions, and in these cases, it can be reassuring if a healthcare professional normalizes these reactions.

Counselling nurse: “Patients often say that when you’re in care, in that cycle, you don’t think about those things so much; you think that you’re safe, you trust that someone is controlling the values and knows what is happening. When you’re not in that system anymore, then you think, ‘oh no, how do I now know what I’m supposed to do, and I feel horrible; I can’t trust that I’m healthy now.’”

During the follow-up phase, many patients are also afraid of a cancer recurrence and questions and contacts with public healthcare can be numerous, which can increase the burden on the system. Here, contacts with the CSF services could help to decrease that burden. Counselling nurses could address the questions that patients may have and also provide psychosocial support to help ease the fear of recurrence.

### Support at cancer recurrence

According to participants, cancer recurrence is a phase during which patients and their close ones usually need a lot of support. Some people have already been using CSF services, and in such cases, the path to contacting support services is smooth. However, not everyone is aware of these services. Counselling nurses and managers hope that public sector staff would once again inform patients about these services at this stage, especially as this can be a very difficult period for many people, who may receive diagnoses of incurable cancer.

Manager: “Yes, I also think this is a very important matter, that it doesn’t have to be professional help, even though I’ve mentioned psychologists and psychiatrists, but equally that the path of care includes peer support services. Some just need peer support, not a psychologist or psychiatrist or anyone else like that—just that peer-group support.”

A recurrence of cancer can, in turn, restart the psychological processing of cancer. For some, it is easier to accept this situation particularly when they have already previously processed the cancer diagnosis. However, some people can have very strong psychological reactions, as their psychological processing must start from the beginning. The reactions of close ones can also vary.

### Support during palliative and end-of-life care and beyond

According to participants, the palliative and end-of-life period is understandably very difficult both for patients and their close ones, and the way that these two groups deal with this difficult situation can vary greatly. In this situation, the service needs are also different for patients and for their close ones, although both need psychosocial support and information.

Counselling nurse: “So, in palliative terminal care, when the person close to you dies, it is also important to have answers, because you have so many questions, like did I do the right thing and did I support them and what about the pain, and why did they end up dying in this way or that way. If the relationship had been discussed with someone, you would know that it is possible to call or visit them and ask because the threshold is then lower.”

During end-of-life care and after the patient’s death, close ones can feel that they have been left alone, as they do not have a care connection to the patient’s healthcare unit. Subsequently, support is often very limited. In addition, psychosocial reactions can be very difficult when everything is over. Close ones do not have a care relationship with the healthcare system. Here, again, participants emphasized the professional and peer support that close ones can receive from the CSF.

**Figure 1.**
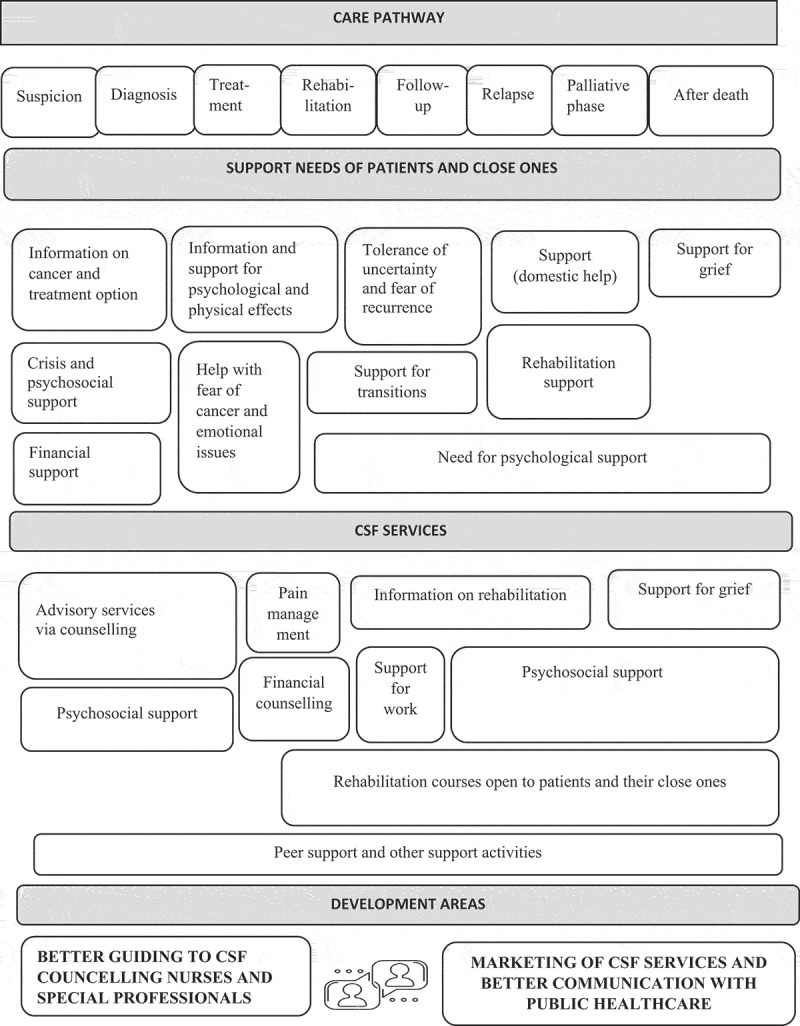
Participants’ perspective on the care pathway, their evaluation of support needs and the role of the CSF

The key results of this study are summarized in Figure 1, showing support services throughout the key phases of the care pathway. Participants emphasized that the CSF can support the public sector’s work throughout the whole patient pathway, according to patients’ and close ones’ individual needs, for example, through psychosocial support, peer support, counselling on benefits and treatment, rehabilitation services, guidance as to the various support services and grief support.

## Discussion

### A key message from the CSF: placing the needs of cancer patients and their close ones at the centre

Participants in this study were experts from cancer societies in Finland with long experience of working with patients, their close ones and their networks. According to the results of this study, these professionals evaluate cancer patients and their close ones as being generally satisfied with the medical treatment for cancer in Finland. However, they emphasize the need for opportunities to reflect their thoughts and for psychosocial support, especially relating to the thoughts and feelings caused by the illness. These results support earlier findings (Evertsen & Wolkenstein, 2010; Molassiotis et al., 2011; Wootten et al., 2014).

According to informants, patients primarily need psychosocial support, discussion with professionals, peer support and information on illness and rehabilitation. Close ones often need information about the disease and its treatment, psychosocial support and discussion with professionals, as previously demonstrated (e.g., Hodgkinson et al., 2007; Lehto et al., 2015; Wootten et al., 2014).

Participants emphasized the variety of support needs and how they differ depending on the phase of the cancer trajectory taking individual needs into account. Patients need a great deal of support once they are diagnosed, during the follow-up and rehabilitation phase, if cancer returns and during palliative care. Close ones also need support during these phases, but they additionally benefit from support after the patient’s death when psychosocial reactions can be very difficult. Close ones do not have a care relationship with healthcare professionals and are often left alone.

However, it was surprising that the treatment phase did not get any predominance in relation to support needed, either in patients or close ones. However, it is obvious that during the treatment phase patients have close contacts with healthcare professionals, and during the follow-up only few visits to the clinic take place. This may cause unmet support needs, and here CSF professionals could provide help and support.

Our results point towards that psychosocial needs are insufficiently addressed in clinical cancer care, and there is a need for collaboration with other service providers, such as cancer associations. The need for information is constant during the illness trajectory. The results also show that knowledge of the CSF services is limited. It is especially important that the CFS and public healthcare professionals inform patients about these services at the time of diagnosis, when the CSF may provide opportunities for discussion and psychosocial support both for those diagnosed and for their close ones. According to participants, many of the needs of patients and their close ones identified in previous studies are being used as part of services provided by the CSF.

Cancer patients and their close ones have received help from a variety of interventions, such as psychotherapy and peer support (Landry-Dattée et al., 2016; Newby et al., 2015), online peer support services (Hartzler et al., 2016), information and support based on empowering interactions (Lundberg et al., 2013) and patient care-related information (Brazil et al., 2014). Some benefit from a variety of technical and social media tools (Yli-Uotila, 2017), which the CSF uses in many ways. Patients could benefit even from as small an amount of support as one phone call a week after diagnosis (Salonen, 2011). However, while information technology can be utilized in developing activities, increasing digitization could run counter to the need expressed by patients and family to meet a professional face to face, as our participants stated.

Participants reflected on the role of public healthcare in Finland. They stressed the important role of public healthcare professionals, who should identify those instances when patients need more support and information and refer them to the CSF and other special professionals, such as social workers, occupational therapists or psychologists. Participants in this study also emphasized the non-simultaneous needs of patients and their close ones as a critical factor in the cancer care pathway, and that it is of critical importance to provide support in a timely fashion. In sustainable cancer care, the focus has to be on what matters most to patients—a principle that should guide the entire cancer care process. One key area for improvement is the marketing of CSF services to healthcare professionals in the public sector, promoting the development of personalized support and, while doing so, better utilizing information technology.

In line with Wait et al. (2017), the needs of cancer patients and their close ones lie at the heart of support services throughout the different stages of the care pathway depicted in Figure 1. Some cancer patients utilize a wide range of services from various specialists (Evans et al., 2015). However, as our results show, unclear operating practices and poor availability of specialist staff are barriers to support for all patients and close ones (e.g., Absolom et al., 2011).

According to participants, the support of close ones is very important for cancer patients, with those patients who receive support from their close ones needing less psychosocial support from professionals, as Heins et al. (2016) also noted. Although close ones can feel worse than the patients, they can be deprived of the help they need because the focus of caring is on the patients (Mosher et al., 2013). According to our study, cancer patients can perceive the diagnosis and its consequences differently from their close ones and have different coping strategies. The timing of needs is non-simultaneous and support, therefore, must be tailored accordingly. For this reason, the CSF should take a more active role in supporting family, as our participants described.

Our participants also talked about the serious financial consequences that cancer can have, as told to them in many discussions with unemployed people, pensioners, single parents and entrepreneurs. Many people do not know what financial benefits they are entitled to, even though some of them have difficulties in paying for food and medicine. According to our participants, patients should receive better guidance on financial support, for example, from social workers—a conclusion that has also been drawn in research into other long-term illnesses (Aaltonen, 2017; Cahallan & Brintzenho-Feszoc, 2015). As a solution, participants suggested that healthcare professionals adopt a more holistic view of patients (Evertsen & Wolkenstein, 2010) and refer them more often to special professionals.

### Looking to the future: third-sector organizations to play a stronger role in the cancer care pathway

In re-organizing services, a stronger role for non-governmental organizations, such as the CSF, has been proposed in Finland (Brax, 2018). Considering the results of this study, we suggest that one way to do this is to have different stakeholders to participate in a thorough discussion on health policy. It is contradictory that in a welfare state such as Finland, people with cancer are left without support and information, while organizations such as the CSF exist and can provide exactly these services. Although the role of the CSF as a major private donor to cancer research has been recognized for years, professionals in the public sector remain somewhat sceptical about co-operation with this kind of third-sector organization. Professionals within the CSF speculated that one reason for this is that their support services are not well known, as shown by Pavolini and Spina (2015). Professionals within the public sector should see the CSF as a co-operative facilitator of their own healthcare work. The identified challenges to this are communication and support during the transitions from one phase of the disease pathway to another and from one service provider to another.

As emphasized in the international policy report as cited in Wait et al. (2017), attention should be focused on continuity of care and on other services. Patients and their representatives as well as patient organizations should have a role in national-level planning including the planning of care pathways. The European Cancer Organization (Aapro et al., 2017) suggests improving innovation in cancer care by using “a whole-patient approach.” This means that the strategy is guided by the patient’s needs and patient-relevant outcomes. Research should be carried out in co-operation with patient organizations to have a better understanding of what clinical and psychosocial needs are unmet. Both the need for continuity of care and co-operation between public healthcare and cancer societies, were clearly expressed also in our study results.

Transitions in the care pathway are also a challenge elsewhere in the world. Therefore, there has been a strong emphasis on the use of care plans (Vanhaecht et al., 2010) to support care management in cancer patients (Baker et al., 2008; Mayer et al., 2014; Michael et al., 2013). Also peers and patient advocates could help in care transitions. The CSF trains its peers, but this is not always sufficient to convince professionals in the public sector of their qualifications. According to Jones and Pietilä (2018), peer supporters know the requirements for their tasks. Cancer patient advocacy aims to strengthen the roles of cancer patients and caregivers together with third-sector organizations and to provide information about the gaps in the system of care (Schear et al., 2015).

Our results suggest a need to develop a care pathway, and they are similar to those of Evans et al. (2015), who reported that integrated care models have demonstrated a range of positive outcomes. The delivery of integrated care requires coordination and collaboration across various organizations, care settings and professionals to ensure that patients receive the right care in the right place at the right time, such as case-managed multidisciplinary team care, organized provider networks and financial incentives. This is in line with our model of the care pathway in which the patient and close ones are treated individually in a multi-professional manner and as economically as possible.

Across European healthcare systems, it is estimated that 20% of spending is currently wasted on ineffective interventions. Ultimately, improved efficiency will contribute to more equal access to, and affordability of, healthcare (Wait et al., 2017). According to our results, along with increased financial challenges, the need for services also increases, especially among the elderly, at least some of whose needs could be fulfilled by non-profit organizations and volunteers.

### Strengths and limitations

The strength of this study is that, to date, very little research has been carried out on the role of non-profit organizations, such as the CSF, in supporting patients, and there is even less research concerning support for the patients’ close ones. Furthermore, our participants, who were all employed by the CSF, had a great deal of experience with cancer patients and their close ones and can, thus, be considered well aware of their needs and concerns. Particular emphasis was given to the regional spread of participants, who were selected from all over Finland. The sample size for this study is considered adequate, and an appropriate qualitative methodology was applied (Gray et al., 2017).

When evaluating the development of a care pathway, it is important to look at it from multiple angles and to make estimates and recommendations using versatile information. This study focused on the views of counselling nurses and managers at the CSF, whose views are considered to be patient-centric because they take into account the whole disease trajectory rather than focusing only on the treatment period, which is understandably the main focus of public healthcare.

This qualitative data obtained from semi-structured group interviews can be considered rich and interesting information, which allowed us to identify important key concepts related to support needs of cancer patients and their close ones. However, these topics need to be further investigated with other methods, including larger questionnaire studies, and there is a need to expand research to other key informants, including patients, close ones and professionals within public healthcare. A care pathway needs to be built in participation with patients and close ones, and these results provide a basis for further collaborative research, which is underway to directly examine the perspectives of these groups as well as healthcare professionals within the public sector.

## Conclusion and implications for the future

According to the professionals in cancer associations, cancer patients and their close ones need more information and support to deal with the disease, including more referrals to specialists, such as those who provide psychosocial and financial support. This should happen right from the beginning of the care pathway. The greatest need for improvement is during the transitions between different stages of the care pathway, at transitions from one service to another and in directing the right people to the right services.

The CSF has an opportunity to provide professional and peer support to patients and their close ones. Such support services are often not sufficiently resourced by the public sector. Participants expressed a willingness to co-operate, and this co-operation should increase both in general and during specific phases of the care pathway.

The results of this study could initiate a dialogue on how we can better identify the needs of patients and their close ones, reduce duplication of work, rationalize care pathways, clarify the role of the third sector and, thus, also generate financial savings. More research is needed on the financial impact of cancer on the patient, on streamlining the care pathway and on the role of non-profit organizations working with the public sector.
